# Performance Evaluation of Styrene-Butadiene-Styrene-Modified Stone Mastic Asphalt with Basalt Fiber Using Different Compaction Methods

**DOI:** 10.3390/polym11061006

**Published:** 2019-06-06

**Authors:** Wensheng Wang, Yongchun Cheng, Peilei Zhou, Guojin Tan, Haitao Wang, Hanbing Liu

**Affiliations:** College of Transportation, Jilin University, Changchun 130025, China; wangws17@mails.jlu.edu.cn (W.W.); chengyc@jlu.edu.cn (Y.C.); whtjlu@163.com (H.W.); liuhb@jlu.edu.cn (H.L.)

**Keywords:** asphalt mixture, styrene-butadiene-styrene, basalt fiber, compaction methods, SGC

## Abstract

Superpave gyratory compaction (SGC) and Marshall compaction methods are essentially designed according to volumetric properties. In spite of the similarity, the optimum asphalt contents (OAC) of the two methods are greatly affected by the laboratory compaction process, which would further influence their performance. This study aims to evaluate the performance of styrene-butadiene-styrene (SBS)-modified stone mastic asphalt (SMA) with basalt fiber by using SGC and Marshall compaction methods. Basalt fiber was proved to improve and strength the basic properties of SBS-asphalt according to test results of asphalt binder. The effects of SGC and Marshall compaction methods on OAC and volumetric properties, i.e., density, air voids (VA), voids in mineral aggregates (VMA), and voids filled with asphalt (VFA), were evaluated in detail. Finally, the pavement performance of asphalt mixture prepared by SGC and Marshall compaction methods were compared in order to analyze the high-temperature creep, low-temperature splitting, and moisture stability performance. Results showed that the OAC of SGC (~5.70%) was slightly lower than that of Marshall method (5.80%). Furthermore, the pavement performance of SGC specimens were improved to a certain extent compared with Marshall specimens, indicating that SGC has a better compaction effect and mechanical performance.

## 1. Introduction

By the end of 2018, the total mileage of highways in China has been 4.846 million kilometers, in which the mileage of expressway was 0.63 million kilometers. Asphalt pavement, as a widely used road facility material all over the world, has been employed with a rapid growing trend for the vigorous developing highway infrastructure construction. Stone matrix asphalt, abbreviated as SMA, is a kind of gap-graded asphalt mixture proposed in Europe in the sixties, which has higher modified asphalt content, fibers, and coarse aggregate, as well as filler [1]. SMA has been almost exclusively used for expressway as surface courses in China because of its superior pavement performance compared to conventional HMA [2,3]. Meanwhile, SMA could provide better durability properties (specifically high rutting resistance) [4,5]. However, from the point of view of pavement performance, many researches on modifications to SMA have been developed in order to design durable pavement infrastructures in our daily lives.

Polymers, as frequently-used asphalt additives, are used for improving the properties of asphalt [6,7,8,9]. Rubber is a kind of polymer and usually recycled from tire rubber. Rubber was early used for modifying asphalt, and rubber-modified asphalt has many advantages: low-temperature cracking resistance, noise reduction, and so on [10]. Thermoplastic polyurethane (TPU), as a reactive polymer, has been studied for the physical performance, such as rheological, thermal, and aging properties. Results showed that TPU could improve aging resistance using Fourier-transform infrared (FTIR) spectroscopy [11]. Among various polymer modifiers, styrene-butadiene-styrene (SBS) has become the most widely used polymer modifier due to its better pavement performance [12,13,14]. Regarding SBS-modified asphalt, Zhang et al. [15] investigated SBS-modified bitumen systematically and the results were analyzed and quantified through fluorescence microscopy. In the light of the existing literatures, SBS can improve the properties of bitumen comprehensively [16,17,18]. This is because adding SBS into asphalt has lots of advantages, such as high resistance to rutting, high flexibility at low temperatures, and reduced adhesion and cohesion failure [19].

Fiber, a kind of strengthening additive, has been extensively employed for bituminous pavement construction, such as traditional lignin fiber, polyester, glass fiber, etc. [20,21,22]. Generally, adding fibers into asphalt mixtures could improve their performance in order to extend service life. Fiber-reinforced asphalt pavements have been employed for various grades of highways in civil infrastructures due to the superior properties. However, from an environmental point of view, a new environmentally friendly fiber—basalt fiber—has attracted a lot of attention due to its good mechanical properties, low water absorption, and high melting point [23]. The authors have worked on basalt fiber reinforced asphalt mixtures, involving the design optimization and freeze-thaw damage evaluation [2,24,25]. The results showed that the addition of basalt fiber into asphalt mixture can improve the pavement performance of asphalt mixtures well.

In addition to the modification of asphalt binders by polymers or the addition of fibers into asphalt mixtures, compaction characteristics are essentially important in affecting the working performance of asphalt mixtures. The Marshall compaction method, as one of the most widely used design methods for asphalt mixtures all over the world, is uncomplicated and inexpensive [26,27]. The traditional Marshall compaction design method using hammer drop impact has some limitations, and cannot effectively simulate the actual pavement compaction situation. However, the exiting literature showed that asphalt mixtures prepared by a superpave gyratory compaction (SGC) method have good correlation with the characteristics of the field core samples from the real pavement [28,29]. Superpave is the abbreviation of superior performing asphalt pavement and it is a new type of bituminous mixture design compared to the Marshall method [30]. The superpave mixture design method, as one product of the Strategic Highway Research Program (SHRP), has taken traffic and climate into account. Zhang et al. [31] investigated the compaction characteristics of various asphalt mixtures with different gradations and asphalt types at three temperatures using CT scanning technology. The results showed that SMA-13 exhibited a better compaction performance and temperature stability than the AC13. Félix et al. [32] evaluated the influences of compaction parameters (i.e., vertical pressure, gyration angles, and compaction temperatures) on volumetric and mechanical properties (i.e., shear stress, density, air voids, and so on) of asphalt mixture. It was found that temperature had more effect on mechanical properties than on compactability for both the SGC and Marshall compaction methods. Gong et al. [33] investigated the influences of coarse aggregates on bituminous mixtures made by SGC through lab testing and modeling in order to analyze the volumetric and densification properties. They found that flat aggregates had a greater influence on the compactability than elongated aggregates.

The main objective of this study is to evaluate the performance of SBS-modified SMA with basalt fiber by using Superpave gyratory and Marshall compaction methods. Firstly, the physical properties of SBS-modified asphalt with or without basalt fiber were measured for comparative analysis. Then, considering the volumetric properties as well as other mechanical parameters, OAC could be determined for the two compaction methods. Furthermore, the pavement performance of SBS-modified SMA with basalt fiber were also studied in order to compare and analyze the compaction effects of Superpave gyratory and Marshall compaction methods.

## 2. Raw Materials and Experimental Methods

### 2.1. Raw Materials

The SBS-modified asphalt with penetration between 60 and 80 (0.1 mm) was used in this study and its technical properties are shown in Table 1, which meet the requirement of the specifications JTG F40-2004 (ASTM D6373-16) (Ministry of Transport of the People’s Republic of China: Beijing, China).

In this study, crushed basalt produced from Jiutai Basalt Industry Co., Ltd., Jilin, China, was used as coarse and fine aggregates, and ground limestone from Siping Quarry Industry Co., Ltd., Jilin, China was adopted as filler. Their physical properties meet the standard requirements (JTG F40-2004), whose technical properties are listed in Table 2, Table 3 and Table 4.

Basalt fiber of 6 mm length was chosen for SBS-modified SMA in this study. The appearance of basalt fiber is golden brown and basalt fiber has good mechanical properties, low water absorption, and high melting point. The detailed technical properties have been given in the previous study [2,24].

### 2.2. Specimen Preparations

**Figure 1:**
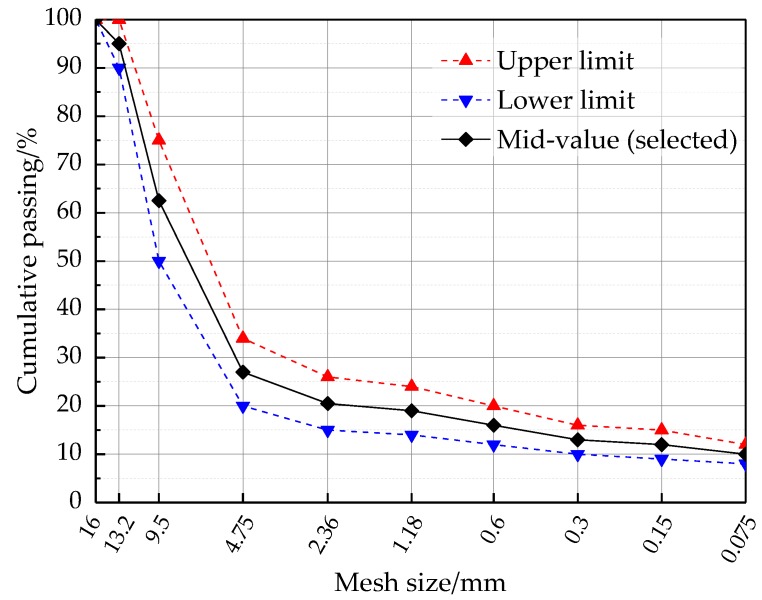
Gradation of SMA-13 in this study.

The asphalt mixture specimens modified by SBS and fiber in this study consisted of SMA with a nominal maximum size of 13.2 mm, which has been extensively applied for pavement infrastructure engineering all over the world because of its high rutting resistance and durability [34]. To avoid the influences of aggregate composition and basalt fiber content on asphalt mixture, the median gradation of SMA-13 was selected for asphalt mixture, as illustrated in Figure 1, which ensures the comparability. Basalt fiber content was chosen as 0.34% by mass of SBS-modified asphalt according to Wang et al. [2]. Five kinds of asphalt compositions (i.e., asphalt-aggregate ratios) were prepared, namely, a range with increments of 0.4% from 4.7% to 6.3%. The aforementioned SBS-modified SMA with basalt fiber was prepared using two compaction methods, i.e., Marshall compactor as well as Superpave gyratory compactor.

#### 2.2.1. Marshall Compaction Method

The Marshall mixture design method is one of the most widely used design methods for asphalt mixtures all over the world, and is simple and inexpensive [26,27]. The Marshall compactor can be used to prepare cylindrical asphalt mixture specimens with 101.6 mm (diameter) × 63.5 mm (height) or 152.4 mm (diameter) × 95.3 mm (height) according to the specification JTG E20-2011 T0702 (ASTM D1559) (Ministry of Transport of the People’s Republic of China: Beijing, China).

**Figure 2:**
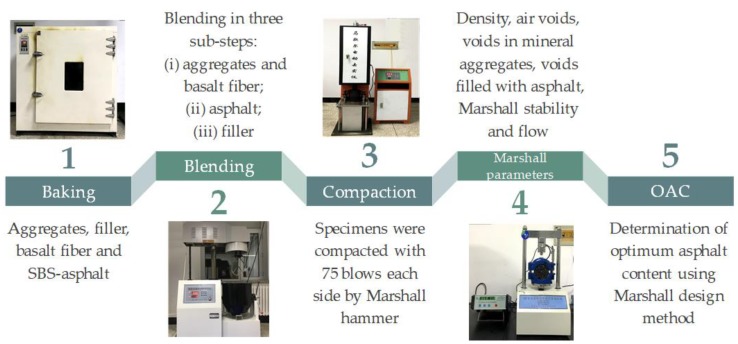
Preparation procedure of styrene–butadiene–styrene-stone mastic asphalt (SBS-SMA) with basalt fiber by Marshall method.

The whole preparation procedure of the standard Marshall mix design method can generally be divided into five stages, as shown in Figure 2, and the corresponding procedure is presented in detail in the following.

- Step 1: Baking. The needed amounts of coarse and fine aggregates, fiber, as well as filler were placed in the oven at 150 °C for 4–6 h and the SBS-modified asphalt was heated to 180 °C.
- Step 2: Blending at a mixing rate of 80 rev/min. The preheated aggregates and basalt fiber were blended together in the mixing pot at 170 °C for 90 s, then the weighted SBS-modified asphalt was poured into the pot and blended for 90 s in order to make aggregates uniformly coated with asphalt binder, finally the preheated filler was added and blended for 90 s again.
- Step 3: Compaction with the Marshall hammer. Each asphalt mixture specimen was compacted by free falling drop with a Marshall hammer of 4536 g sliding weight. The number of blows was 75 and the specimen was then turned over for repeated compaction.
- Step 4: Marshall design parameters. The parameters generally include volumetric properties (i.e., density, air voids, voids in mineral aggregates, and voids filled with asphalt) as well as Marshall stability and flow.
- Step 5: Optimum asphalt content (OAC). OAC was finally determined on the basis of the comprehensive results of Marshall parameters and volumetric analysis.

All the volumetric properties of SMA specimens were conducted and measured at the room temperature of 25 ± 0.5 °C. In accordance to the specification JTG E20-2011 T0706 (ASTM D2726), the density of specimens (i.e., bulk specific density (ρ_f_)) can be measured through weighting SMA specimens in air and water, respectively, and can calculated by Equations (1) and (2). According to the specification T0705 (ASTM D3203), *VA*, *VMA*, and *VFA* can be obtained by using the vacuum sealing method, which could be calculated by Equations (3)–(5).

γ_f_ = *m*_a_/(*m*_f_ − *m*_w_) (1)

ρ_f_*=* γ_f_*×* ρ_w_ (2)

*VA* = [1 − γ_f_/γ_TMD_] × 100 (3)

*VMA* = [1 − γ_f_ × *P*_s_/γ_sb_] × 100 (4)

*VFA* = [(*VMA* − *VA*)/*VMA*] × 100 (5)

where γ_f_ represents bulk specific gravity, γ_TMD_ represents theoretical maximum specific density, *P*_s_ represents the aggregate content percent by weight of mixture, and γ_sb_ represents bulk specific gravity of aggregates.

Afterwards, in accordance with specification JTG E20-2011 T0709 (ASTM D6927), the *MS* test can be carried out for the SMA Marshall specimens. The prepared Marshall specimens were placed into water bath at 60 °C for 0.5 h to guarantee the uniform temperature distribution inside the specimens. Then, the *MS* and *FL* tests were performed by employing the Marshall stabilization test equipment with a loading rate of 50 mm/min. Until the SMA specimens reached failure, the failure force and deformation were regarded as *MS* and *FV*, respectively.

#### 2.2.2. Superpave Gyratory Compaction Method

**Figure 3:**
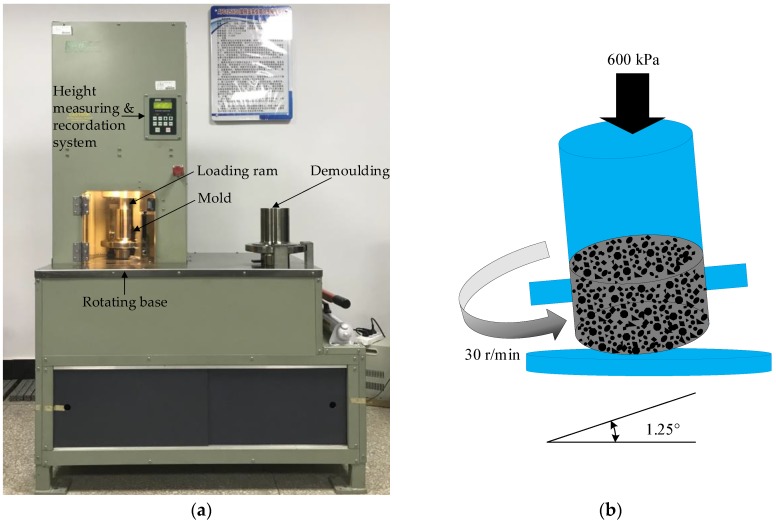
Superpave gyratory compaction: (**a**) SGC test and (**b**) diagram of SGC.

The superpave gyratory compactor could better reflect the actual pavement service conditions, such as temperature, vehicles, and so on, in order to meet the requirements of simulative compaction and traffic loading. The procedure of superpave mix design method is similar to that of Marshall mix design method, consisting of five basic steps:

- Steps 1 & 2: Baking and blending. These two steps of the superpave mix design method are the same as the Marshall mix design method.
- Step 3: Superpave gyratory compaction. Compaction temperature and gyratory compactor parameters were firstly determined, as shown in Figure 3. The SGC specimens with 150 mm diameter were compacted at 160 °C, and the designed gyratory number was set as 100 under the pressure of (600 ± 18) kPa, gyration angle of (1.25 ± 0.02)°, and gyration speed of 30 r/min. After compaction, the SGC specimens were extruded from the mold and allowed to cool.
- Step 4: Density and voids calculations. The test parameters (i.e., density, *VA*, *VMA*, and *VFA*) were measured for further determining OAC.
- Step 5: Optimum asphalt content (OAC). The OAC of SGC asphalt mixtures was finally determined based on the comprehensive results of density and voids analysis.

In the superpave mix design method, the level of compaction of SGC specimens is a function of gyratory compaction number, reflecting the stability of construction and service of road. According to the traffic level designed by equivalent single axle load (ESAL) of AASHTO R35-04, the designed gyratory compaction number (*N*_des_) was selected as 100, which is usually used to investigate the effect of gyratory compaction; the initial gyratory compaction number (*N*_ini_) was 8 to simulate the compaction effect of paving; and the maximum gyratory compaction (*N*_max_) was 160, which is equivalent to the effect of lasting action of traffic loads. Generally, the relationship curve between compaction level and gyratory compaction number could be divided into two phases. The phase of *N*_ini_→*N*_des_ is used to analyze the compaction characteristic during construction (i.e., paving and rolling), and the phase of *N*_des_→*N*_max_ is used to analyze the compaction characteristic during service (i.e., traffic loading). Hence, the compaction characteristic of *N*_des_→*N*_max_ was considered and investigated in this paper.

Each group of different asphalt contents prepared three parallel specimens, and the volumetric properties of SGC specimens were measured and summarized as the average values of three specimens after the designed gyratory compaction number (*N*_des_ = 100). Under the designed gyratory compaction number of *N*_de*s*_ = 100, VA, VMA, and VFA can be calculated according to Equations (5)–(7).

*VA* = [(*G*_mm_ − *G*_mb_)/*G*_mm_] × 100% (6)

*VMA* = [1 − (*G*_mb_ × *P*_s_)/*G*_sb_] × 100% (7)

where *G*_mm_ is the theoretical maximum density of SGC specimens using AASHTO T209, *G*_mb_ is the bulk specific gravity of SGC specimens using AASHTO T166, and *G*_sb_ is the bulk specific gravity of the total aggregate.

The level of compaction at *N*_des_ = 100 and *N*_ini_ = 8 is defined as

*% G*_mm_*@N*_des_ = *G*_mb_/*G*_mm_ × 100% (8)

*% G*_mm_*@N*_ini_ = *% G*_mm_*@N*_des_ × [*H*_des_/*H*_ini_] (9)

Subsequently, the *% G*_mm_ at any number of gyratory compaction number (*N*_x_) could be calculated by multiplying *% G*_mm_ *@ N*_des_ by the ratio of the heights at *N*_des_ and *N*_x_.

Besides, the curve of phase *N*_ini_→*N*_des_ usually presents a semilogarithmic function and the slope of this curve (*K*_1_) is used to represent the rate of gyratory compaction as well as to evaluate the compaction characteristics. The *K*_1_ value is defined by Equation (10):

*K*_1_ = (*% G*_mm_*@N*_des_ − *% G*_mm_*@N*_ini_)/(ln*N_d_*_es_ − ln*N*_ini_) (10)

### 2.3. Experimental Methods

#### 2.3.1. Testing Procedure of Asphalt Binder with Basalt Fiber

The previous literatures showed that the performance of asphalt mixtures is related to the adhesion and machinal performance of asphalt [35,36]. In asphalt mixtures, asphalt binder can not only be used to cohere coarse and fine aggregates, but may also play a role in stabilizing aggregates. Generally, fiber materials are adopted for asphalt materials in order to reinforce and crack resistance as well as enhance the bonding strength of asphalt binder [37]. Therefore, several basic performance tests of the asphalt binder with and without basalt fiber were carried out for the comparative study of the reinforcement of basalt fiber.

The cone penetration test (CPT), proposed by Chen et al. [38], is an experimental means to measure the shearing strength of modified asphalt with fiber. Based on the equilibrium equation of force, the shear stress (τ) of asphalt can be obtained by solving Equation (11):

τ = [981*Q*cos^2^(α/2)]/[π*h*^2^tan(α/2)] (11)

where *Q* = 150 g, *h* is the sink depth, and α = 30°.

**Figure 4:**
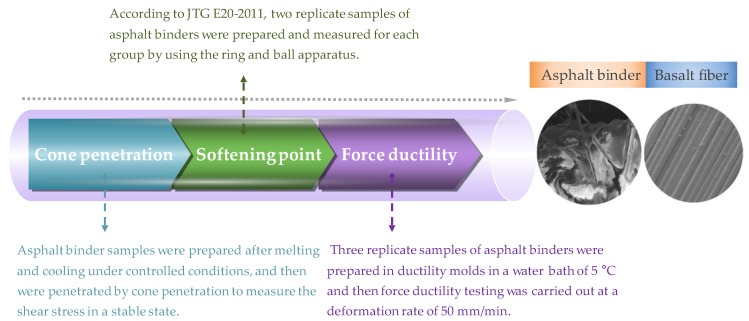
Testing procedure of asphalt binders.

Softening point test (SPT) is the elementary and commonly used experimental technique for the sake of evaluating the high-temperature susceptibility of bitumen and can be obtained according to JTG E20-2011. With respect to the ductility test (DT), it was carried out at 5 °C and the maximum force and stretched elongation were recorded. The corresponding testing procedure is shown in Figure 4.

#### 2.3.2. Pavement Performance Tests of Asphalt Mixture with Basalt Fiber

##### Uniaxial Static Compression Creep Test

**Figure 5:**
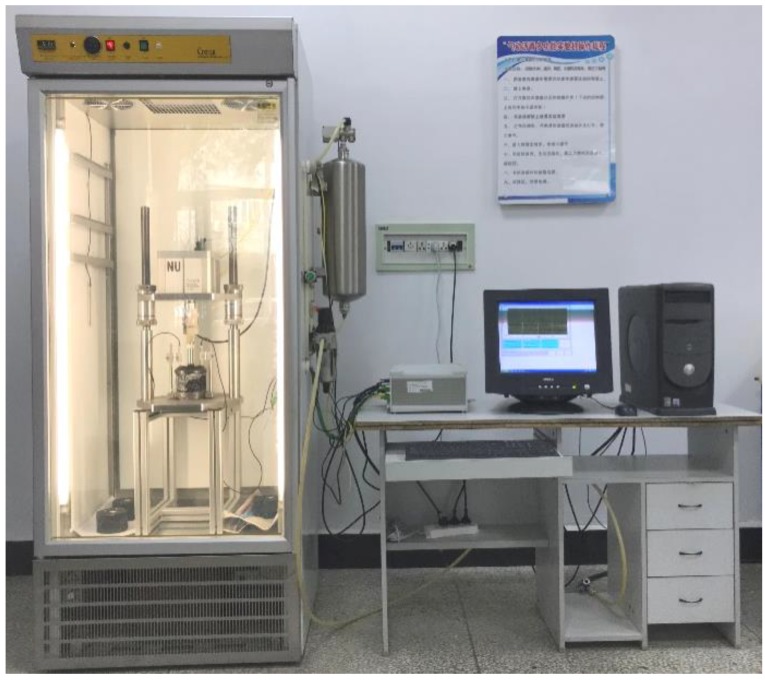
Uniaxial static compression creep test in this paper.

Uniaxial static compression creep test is a common experimental method for investigating the high-temperature creep performance of asphalt mixtures, which was carried out by using a servo-pneumatic universal testing machine (NU-14, Cooper Technologies Ltd, Ripley, UK) as shown in Figure 5. Asphalt mixture specimens prepared by Marshall and Superpave gyratory compaction methods were placed in the temperature chamber of 50 °C for 5 h to ensure uniform internal temperature. Then, by imposing vertical constant loading on asphalt mixture specimens and measuring the deformation of specimens under the constant stress by means of linear variable differential transformers (LVDTs), the stress–strain creep curve of specimens can be calculated.

##### Low-Temperature Splitting Test

**Figure 6:**
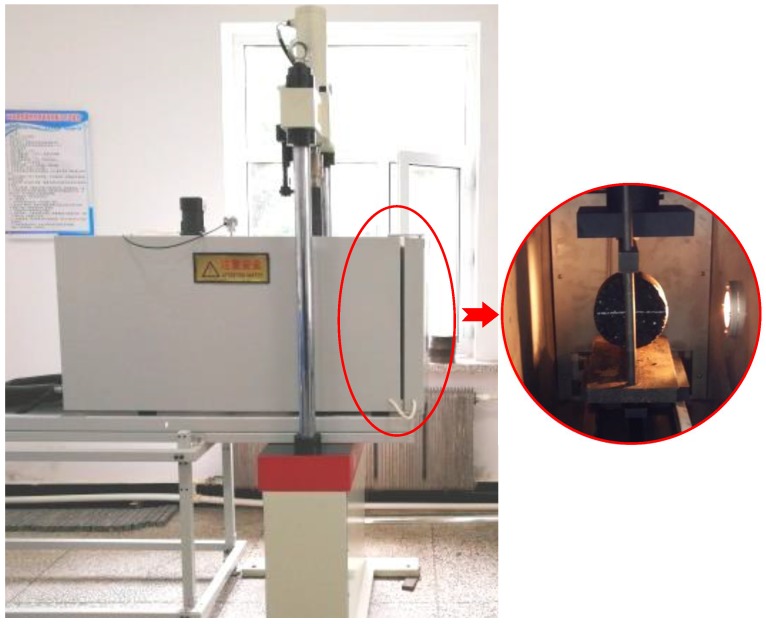
Splitting test in this paper.

It was proved that basalt fiber could improve the low-temperature crack resistance of asphalt mixture [24]. According to the specification JTG E20-2011 T0716, the low-temperature crack resistance of asphalt mixture specimens was carried out by an electro-hydraulic servo material testing machine with the maximum force of 100 kN (shown in Figure 6). This testing machine can control the internal temperature in chamber and its accuracy is 0.01 kN. Specimens were laid into the controller of −10 °C for 6 h to ensure uniform internal temperature; we then applied a force at constant velocity of 1 mm/min.

##### Moisture Stability Test

Following JTG E20-2011-T0729 based on AASHTO T283, the freeze-thaw splitting strength ratio was adopted to study and access the moisture stability performance of asphalt mixtures prepared by different compaction methods. In this study, the testing procedure was similar to the low-temperature splitting test; a detailed description has been introduced in the previous study [26].

## 3. Result and Discussion

### 3.1. Physical Properties of SBS-Modified Asphalt Binder Reinforced with Basalt Fiber

**Figure 7:**
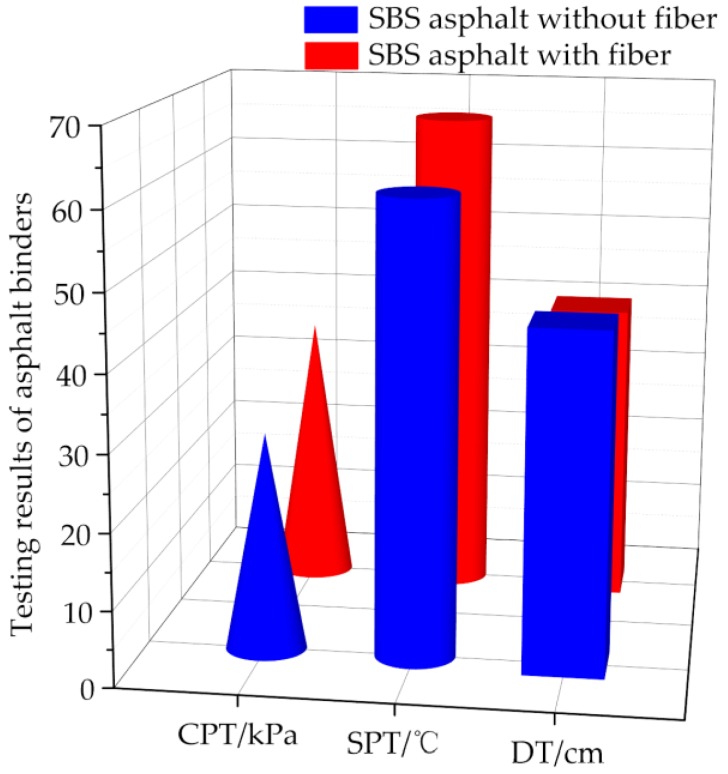
Uniaxial static compression creep test in this paper.

The physical results of CPT, SPT, and FDT of SBS-modified asphalt binder incorporating basalt fiber are illustrated and compared with asphalt binder without basalt fiber in this section, as shown in Figure 7.

Regarding the CPT result in Figure 7, it can be clearly seen that adding basalt fiber into asphalt could improve its shear strength. This is because a spatial networking structure formed by basalt fiber in asphalt would play a role of reinforcement. Meanwhile, it was also proved that basalt fiber could also absorb the light components of asphalt to improve the viscosity [37,39]. As plotted in Figure 7, the softening point of the asphalt binder incorporating basalt fiber was slightly higher than that of asphalt binder without basalt fiber, which is due to a weaker temperature susceptibility of asphalt binder induced by basalt fiber. This may be also due to the reinforcement effect of basalt fiber, leading to higher stiffness and bitumen absorption. The ductility result of asphalt binders with/without basalt fiber at 5 °C were illustrated in Figure 7. It was found that the ductility of asphalt binder with basalt fiber was slightly weaker than that of asphalt binder without basalt fiber. Due to the spatial networking structure of basalt fibers in asphalt, it may reach failure at a larger tensile force, while the failure cross section of asphalt binder samples would be rough, easily resulting in tensile failure.

### 3.2. Optimum Asphalt Content of Modified Asphalt Mixture Using Different Compaction Methods

#### 3.2.1. Marshall Compaction Method

**Figure 8:**
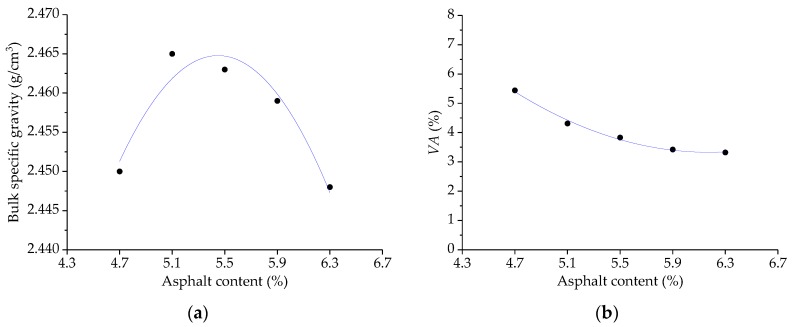

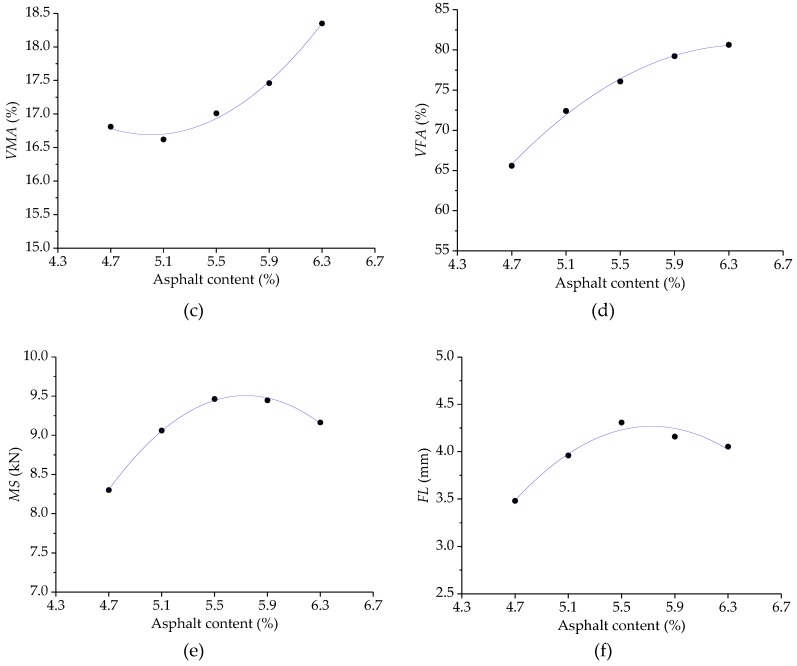
The Marshall mix design results of asphalt mixture with basalt fiber: (**a**) Bulk specific gravity, (**b**) *VA*, (**c**) *VMA*, (**d**) *VFA*, (**e**) *MS*, and (**f**) *FL*.

A series of Marshall asphalt mixtures of SMA-13 gradation with asphalt-aggregate ratios of 4.7%, 5.1%, 5.5%, 5.9%, and 6.3% were prepared, in which three specimens were made for each group. Next, these Marshall specimens were tested and recorded on the basis of the standard Marshall mixture design method. The variation results of bulk specific gravity, *VA*, *VMA*, *VFA*, *MS*, and *FL* with asphalt-aggregate ratios are given in Figure 8. Therefore, the OAC of SBS-SMA containing basalt fiber can be determined following the Marshall design method based on the comprehensive consideration of the maximum bulk specific gravity, maximum Marshall stability, as well as target air voids [24]. In general, due to the fiber’s absorption of asphalt, the OAC of asphalt mixture with basalt fiber would increase [37,38,39]. For the Marshall asphalt mixtures, the OAC value was determined as 5.80% by weight of aggregates. Meanwhile, the test result meets the Marshall design requirements.

#### 3.2.2. Superpave Gyratory Compaction Method

**Figure 9:**
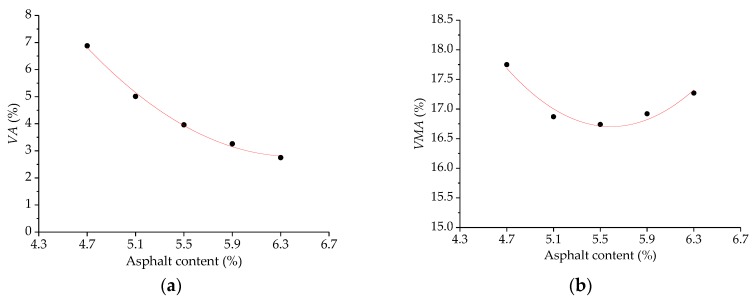

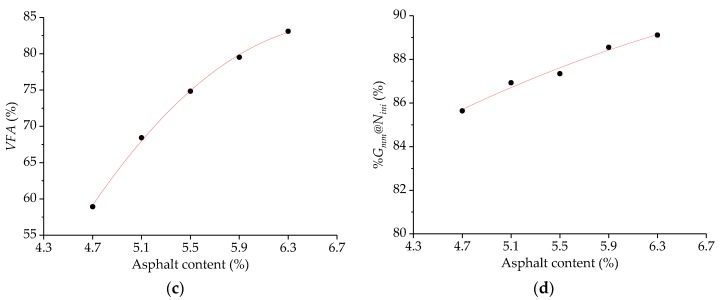
The Superpave mix design results of asphalt mixture with basalt fiber: (**a**) *VA*, (**b**) *VMA*, (**c**) *VFA*, and (**d**) % *G*_mm_*@N*_ini_.

Similarly, three replicate SGC specimens were prepared for several asphalt-aggregate ratios of 4.7%, 5.1%, 5.5%, 5.9%, and 6.3%. Next, the prepared specimens were measured for the maximum theoretical specific gravity (*G*_mm_). According to Equations (8) and (9), % *G*_mm_*@N*_ini_ can be calculated by multiplying *% G*_mm_*@N*_des_ by the ratio of the height at *N*_des_. The results of *VA*, *VMA*, *VFA*, and *% G*_mm_*@N*_des_ are plotted in Figure 9. Results indicate that the variations of the volumetric properties of SGC specimens are similar to those of Marshall specimens. The results of *% G*_mm_*@N*_des_ are approximately proportional to the asphalt-aggregate ratio, and are less than 89% within the scope of the specification.

**Figure 10:**
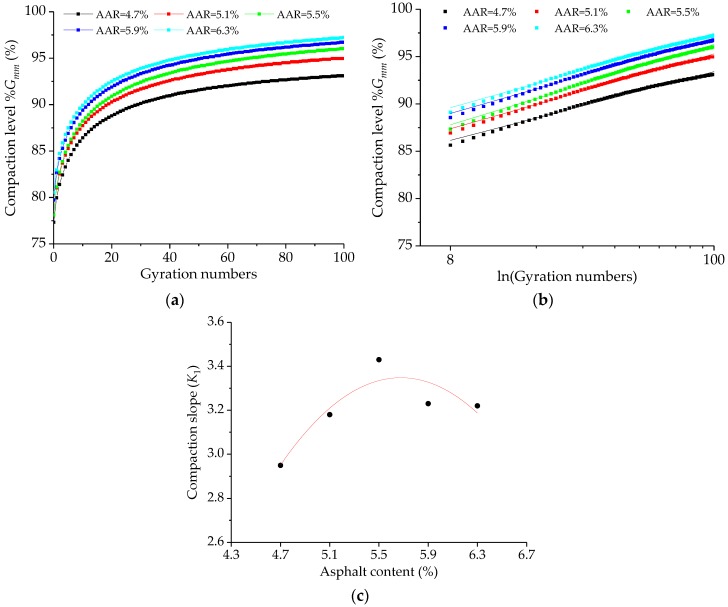
The compaction characteristics of SGC specimens with basalt fiber: (**a**) compaction curve, (**b**) compaction level during *N*_ini_→*N*_des_, and (**c**) *K*_1_ value.

The compaction curve of asphalt mixture SMA-13 at different asphalt-aggregate ratios can be obtained during the compaction period. And the compaction level would be also calculated and illustrated in Figure 10a based on the SGC test. Meanwhile, for analyzing the phase of *N*_des_→*N*_max_, Figure 10b shows the compaction level versus the logarithm of gyration numbers, which can be fitted linearly. It is clearly illustrated that the compaction level increases with the increases of asphalt-aggregate ratio at the same compaction parameters. By comparing the results of the Marshall and Superpave compaction methods, the volumetric properties of these specimens are similar to each other.

Besides, the slope of the compaction curve (*K*_1_) is employed to evaluate the compaction characteristics in this study. By Equation (10), the slopes of the compaction curves (*K*_1_) are calculated, and Figure 10c shows the slope result of *K*_1_ of SGC specimens with the identical SMA-13 gradation but with various asphalt-aggregate ratios. In general, a larger *K*_1_ value indicates that the asphalt mixture is easier to be compacted during construction. On the contrary, it is difficult for a smaller *K*_1_ value to compact in the construction process, which is not conducive to construction. From Figure 10c, the *K*_1_ value increase initially and then decreases with increasing asphalt-aggregate ratio. This result would be attributed to the friction among particles in asphalt mixtures. Under the case of asphalt content less than the OAC, the friction among particles in asphalt mixtures decreases with increasing asphalt content, which is easier to achieve compaction effect. Nevertheless, when the asphalt content is over than the OAC, the thickness of bitumen film reaches a certain value. It cannot significantly improve the compaction characteristics of asphalt mixtures by increasing asphalt content, and even has some opposite effects. Therefore, the OAC value of 5.70% by weight of aggregates is recommended for the SGC specimens of SMA-13 gradation based on the comprehensive consideration of the volumetric properties and compaction characteristics. Meanwhile, the test result can meet the superpave design requirements.

### 3.3. Comparative Analysis of Pavement Performance of Asphalt Mixtures by Different Compaction Methods

The pavement performance of asphalt mixtures is generally closely related to their compaction process. The traditional Marshall mix design method through the drop hammer impact has some limitations and could not be effectively simulate the actual pavement compaction situation. Nevertheless, the previous literatures showed that the asphalt mixture specimens prepared by Superpave gyratory compaction method have a good correlation with the characteristics of the field core samples from the real pavement. In Section 3.2, the OACs of asphalt mixtures prepared by Marshall and Superpave gyratory compaction methods have been determined according to the compaction characteristics and volumetric properties. For the sake of further evaluating the performance of asphalt mixtures prepared by two compaction methods, the high-temperature static creep, low-temperature splitting, and freeze-thaw splitting tests were selected and conducted for high- and low-temperature as well as moisture stability performance.

#### 3.3.1. High-Temperature Creep Performance

The suitable uniaxial compression stress level was firstly determined based on compression failure strength of asphalt mixture specimens. The specimens of the two kinds of compaction methods had an external force applied to them at constant speed of 2 mm/min in a temperature-controlled chamber set to 50 °C. The compressive strength of Marshall specimens was ~1.72 MPa and that of the SGC specimens was 2.01 MPa. The vertical constant loading for uniaxial compression static creep test is usually determined as 0.1 times of the compressive strength, so that the applied stress level of the high-temperature creep test was kept as 170 kPa for 1800 s; the resulting stress recovery was 180 s.

**Figure 11:**
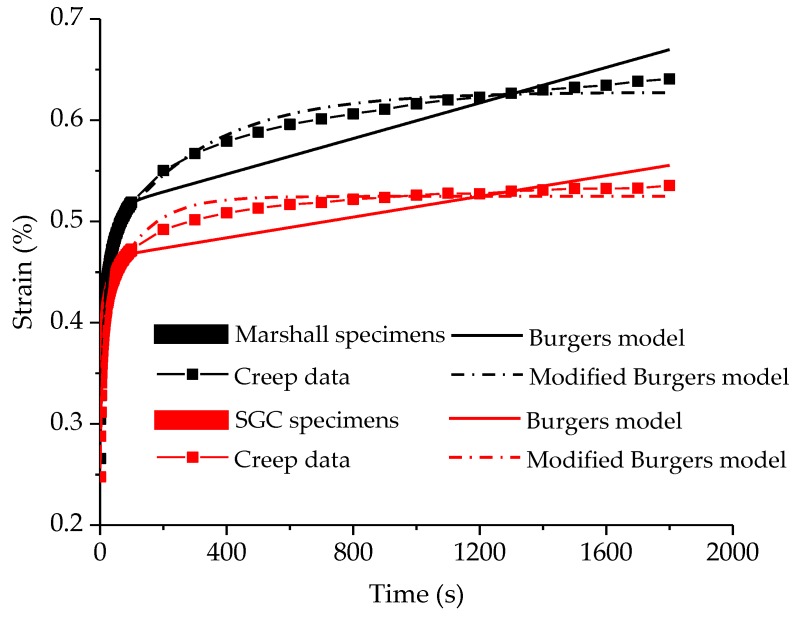
Comparative result of the high-temperature creep test.

**Figure 12:**
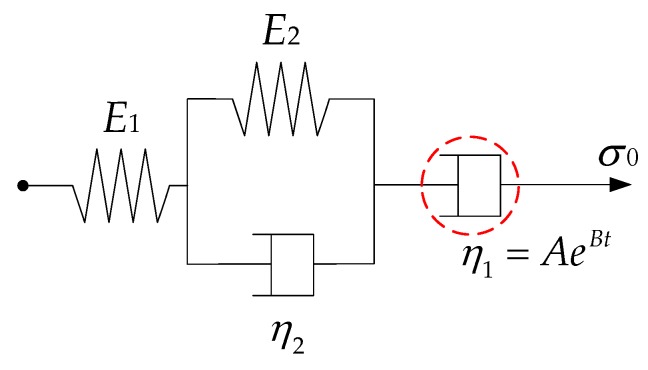
Schematic representation of Burgers and modified Burgers models.

Figure 11 shows the creep curves of Marshall and SGC specimens. Obviously, there is a clear difference between the two curves of Marshall and SGC specimens. A higher creep strain generally implies a poorer deformation resistance. Thus, it could be considered that the Superpave gyratory method can improve the deformation resistance in contrast to the Marshall compaction method. Besides, the creep result of these two specimens are further analyzed by using Burgers model and modified Burgers model. The Burgers model (as plotted in Figure 12) is a series combination of Maxwell and Kelvin models, indicating elastic and viscoelastic deformation, viscous flow, and so on [40,41].

The creep function versus time for Burgers model is given by

(12) $$\varepsilon (t)=\sigma_{0}\left[\frac{1}{E_{1}}+\frac{t}{\eta_{1}}+\frac{1}{E_{2}}\left(1-e^{-E_{2}t/\eta_{2}}\right)\right]$$

where *E*_1_, *E*_2_, *η*_1_, and *η*_2_ are viscoelastic constants obtained by fitting function.

The creep function versus time for modified Burgers model is given by

(13) $$\varepsilon (t)=\sigma_{0}\left[\frac{1}{E_{1}}+\frac{\left(1-e^{-Bt}\right)}{AB}+\frac{1}{E_{2}}\left(1-e^{-E_{2}t/\eta_{2}}\right)\right]$$

where *A* and *B* are also viscoelastic constants determined by fitting function.

Figure 11 also illustrates the fitting result of Burgers and modified Burgers models for Marshall and SGC specimens, in which the detailed fitting parameters are listed in Table 5 and Table 6. The fitting coefficients (*R*^2^) are more than 0.96, meaning that the fitting models could represent the static creep features of Marshall and SGC specimens well. With regard to the viscoelastic parameters, *E*_1_ is the modulus of immediate elasticity model, *E*_2_ is the modulus of delayed elasticity model, *η*_1_ is the coefficient of viscosity of the Burgers model and related permanent deformation after unloading, and the retardation time is defined as τ = *η*_2_/*E*_2_. Thus, the high-temperature creep characteristics of Marshall and SGC specimens can be compared and analyzed based on these viscoelastic parameters. According to the comparative result in Table 5 and Table 6, for either the Burgers model or modified Burgers model, the *E*_1_ values of SGC specimens are larger than those of the Marshall specimens. This means that SGC specimens have a better resistance to deformation on loading and restore ability after unloading. The result of *η*_1_ illustrate that SGC specimens have higher *η*_1_ values than Marshall specimens, indicating SGC specimens would have a less permanent deformation. Besides, a higher value of *τ* indicates that the asphalt material is close to viscous deformation and the recovery time of viscoelastic deformation after unloading is longer. This would be also proved that Superpave gyratory compaction method could improve the deformation resistance of asphalt mixtures. Meanwhile, Figure 11 indicates that the modified Burgers model can represent the viscoelastic performance of asphalt mixtures.

#### 3.3.2. Low-Temperature Splitting Test

**Figure 13:**
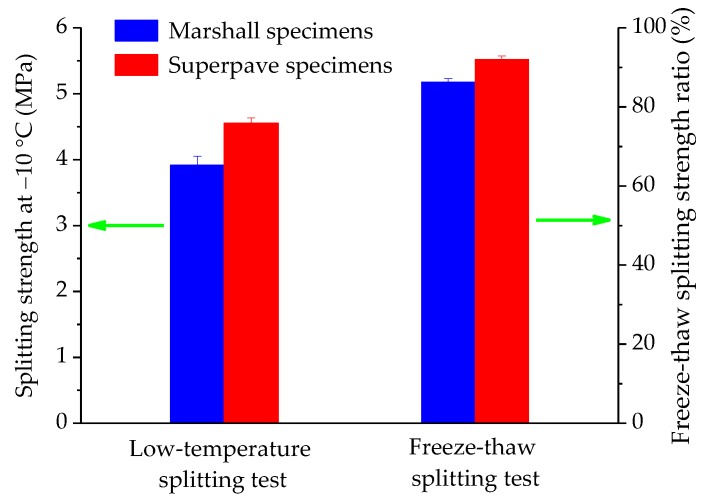
Comparative result of the low-temperature and freeze-thaw splitting tests.

In cold regions, asphalt mixtures have the risks of cracking when subjected to traffic and environmental factors. The low-temperature splitting test is a common test for the sake of illustrating the low-temperature characteristic, and the splitting strength result of −10 °C are used for characterizing the tensile resistance. The low-temperature splitting strength result for these two compaction specimens are presented in Figure 13. Result indicate that SGC specimens have a better low-temperature cracking resistance than Marshall specimens. This result could be attributed to the good compaction characteristics of Superpave gyratory compaction method.

#### 3.3.3. Freeze-Thaw Splitting Test

Moisture susceptibility of asphalt mixtures is one of the most common performance factors evaluated. The experimental process is similar to the low-temperature and specimens need to be pretreated under the freeze-thaw cycle at −18 and 60 °C. The freeze-thaw splitting ratio result of Marshall and SGC specimens are detailed in Figure 13. It can be seen that SGC specimens have better moisture stability than Marshall specimens. This result would be also attributed to the better compaction characteristics of Superpave gyratory compaction method. In general, moisture susceptibility of asphalt mixture is related with the combination between asphalt and aggregates. The OAC of SGC specimens is slightly lower than that of Marshall specimens, and the compaction process of SGC is superior comparing with Marshall method. Therefore, it could be easily understood that SGC specimens have better moisture stability.

## 4. Conclusions

This paper evaluated the performance of SBS-modified SMA specimens with basalt fiber using Superpave gyratory and Marshall compaction methods. The effect of basalt fiber in asphalt was firstly studied based on cone penetration, softening point and force ductility tests. Subsequently, two kinds of asphalt mixtures were prepared by using Superpave gyratory and Marshall compaction methods to determine the OAC based on the volumetric properties. Finally, the pavement performance of Superpave and Marshall SMA specimens were also compared and analyzed according to the high-temperature creep, low-temperature splitting, and freeze-thaw splitting tests. Thus, the above experimental findings were presented as follows.

- Due to the addition of basalt fiber, the shear strength and viscosity of modified asphalt were improved, while the force ductility result slightly decreased. This could be attributed to the spatial networking structure of basalt fibers in asphalt.
- The OACs of two kinds of asphalt mixture specimens were determined according to the volumetric properties, in which the OAC was recommended as 5.80% for Marshall specimens and 5.70% for SGC specimens, respectively. The slight difference of OACs may be due to the compaction procedures.
- The slope of compaction curve (*K*_1_) increase initially and then decreases with increasing asphalt-aggregate ratio, indicating that the compaction effect become better firstly and then worse. This is because the friction among aggregates decreased with asphalt content, however, it would bring in some opposite effects when asphalt content is beyond the OAC.
- Due to the better compaction characteristics of SGC, the mechanical performance of SGC asphalt mixtures were improved to a certain extent. The larger *E*_1_ and smaller *η*_1_ illustrate that SGC specimens have a better resistance to deformation compared with Marshall specimens. Meanwhile, the better compaction characteristics also lead to better low-temperature and moisture stability performance.

## Figures and Tables

**Table 1:** Technical properties of SBS-modified asphalt.

Test Items		Standards	Values	Requirements
Penetration	0.1 mm (@ 25 °C, 100 g, 5 s)	T0604	71	60~80
Ductility	cm (@ 5 °C, 5 cm/min)	T0605	45	≥30
Softening point	°C	T0606	60.5	≥55
Density	g/cm^3^	T0603	1.018	—
Flash point	°C	T0611	262	≥230
RTFOT				
Mass loss	%	T0609	−0.094	±1.0
Penetration ratio	% (@ 25 °C)	T0609	66.9	≥60
Ductility	cm (@ 5 °C)	T0605	33.2	≥20

**Table 2:** Technical properties of basalt coarse aggregates.

Test Items			Values	Requirements
Crushing value		%	13.6	≤26
Los Angeles abrasion value		%	17.9	≤28
Apparent specific gravity	13.2 mm	—	2.836	≥2.6
	9.5 mm		2.805	
	4.75 mm		2.726	
Water absorption	13.2 mm	%	0.6	≤2.0
	9.5 mm		0.28	
	4.75 mm		0.7	
Soundness		%	5	≤12
Elongated particle content		%	9.2	≤15
Passing 0.075 mm sieve		%	0.3	≤1

**Table 3:** Technical properties of basalt fine aggregates.

Test Items		Values	Requirements
Apparent specific gravity	—	2.723	≥2.5
Water absorption	%	0.64	—
Angularity	s	39.9	≥30
Sand equivalent	%	68	≥60

**Table 4:** Technical properties of limestone mineral filler.

Test Items			Values	Requirements
Apparent density		t/cm^3^	2.712	≥2.5
Hydrophilic coefficient		—	0.63	<1
Water content		%	0.3	≤1
Plastic index		%	2	<4
Granular composition	<0.6 mm	%	100	100
	<0.15 mm		92.5	90~100
	<0.075 mm		81.8	75~100

**Table 5:** Fitting parameters of Burgers model.

Types	*E*_1_ (MPa)	*E*_2_ (MPa)	*η*_1_ (MPa·s)	*η*_2_ (MPa·s)	τ (s)	*R* ^2^
Marshall specimen	50.9	80.9	182168	1867.5	23.1	0.970
SGC specimen	55.5	91.3	312878	1606.2	17.6	0.962

**Table 6:** Fitting parameters of modified Burgers model.

Types	*E*_1_ (MPa)	*E*_2_ (MPa)	*η*_2_ (MPa·s)	*A* (MPa)	*B* (MPa)	*R* ^2^
Marshall specimen	55.8	100.9	30567.8	1135.5	0.07698	0.991
SGC specimen	66.6	132.1	15011.6	6351.8	0.15401	0.992
